# Genomic and epidemiological insights into the emergence and dominance of MRSA clones in Riyadh’s healthcare facilities

**DOI:** 10.1038/s41598-025-34001-7

**Published:** 2026-01-06

**Authors:** Dalal M. Alkuraythi, Manal M. Alkhulaifi, Dina A. Altwiley, Mohammed Alarwi, Mohammed I. Mujallad, Mohammad K. Alshomrani, Takashi Gojobori, Sulaiman M. Alajel

**Affiliations:** 1 Department of Biological Sciences, College of Science, University of Jeddah, Jeddah, 23445 Saudi Arabia; 2 Department of Botany and Microbiology, College of Science, King Saud University, Riyadh, 11451 Saudi Arabia; 3 Computational Bioscience Research Center, Biological and Environmental Sciences and Engineering, King Abdullah University of Science and Technology, Thuwal, 23955 Saudi Arabia; 4 Food and Drug Authority, Jeddah, 22235 Saudi Arabia; 5 Microbiology Department, Riyadh Regional Laboratory, Ministry of Health, Riyadh, 12746 Saudi Arabia; 6 National Livestock and Fisheries Development Program, Head of Biotechnology Sector, Riyadh, Saudi Arabia

**Keywords:** MRSA, ST5, ST6, Antimicrobial resistance, Genomic epidemiology, Saudi arabia, Genetics, Microbiology, Molecular biology

## Abstract

The emergence and expansion of methicillin-resistant *Staphylococcus aureus* (MRSA) clones in healthcare facilities pose a significant public health concern due to their adaptability and resistance to commonly used antibiotics. In this study, the genomic and epidemiological characteristics of 81 MRSA isolates collected between February and June 2022 were analyzed. ST5 (25.9%) and ST6 (19.7%) emerged as the dominant sequence types, collectively accounting for 45.6% of infections. Whole-genome sequencing and phenotypic analyses revealed that ST5 clone exhibited a broader multidrug-resistant profile compared to ST6 clone, with higher prevalence of β-lactam, tetracycline, and trimethoprim resistance. ST6 clone showed more variable resistance, including aminoglycoside and macrolide genes, suggesting a possible community origin before adaptation to hospital settings. Phylogenetic analysis demonstrated ongoing microevolution within Clonal Complex 5 (CC5), including the identification of novel single-locus variants such as ST8111. Correlation analyses highlighted significant associations between key resistance genes and their phenotypic profiles, reflecting complex co-resistance mechanisms. Additionally, virulence profiling revealed that ST5 uniquely carried *tsst*-1, whereas PVL genes were absent in both ST5 and ST6 and appeared only in a small subset of other MRSA clones. The emergence of highly resistant clones like ST672 underscores the potential concern for sustained genomic surveillance. The observed clonal shift from ST239 to ST5 and ST6 signals a dynamic MRSA landscape in Saudi Arabia, emphasizing the need for integrated molecular epidemiology and targeted infection control strategies.

## Introduction

Methicillin-resistant *Staphylococcus aureus* (MRSA) is a highly adaptable, pathogenic bacterium that poses a serious public health risk^1^. It has the potential to cause severe infections and is resistant to common antibiotics, making treatment more challenging^2^. The emergence and expansion of MRSA clones in healthcare facilities underscores the urgent need for comprehensive epidemiological surveillance and intervention strategies^3^. In Saudi Arabia, studies indicate a recent concerning increase in MRSA diversity, with 18 distinct clonal complexes (CC) identified among MRSA isolates in Riyadh^4^. These include notable new strains such as CC152 and CC361^4^. Previous analyses further elucidated the prevalence of established clonal complexes like CC80, CC6, and CC5, which were predominant from 2009 to 2015, alongside the emergence of novel sequence types (STs) such as ST8-MRSA-IV and ST72-MRSA-IV^5^.

The prevalence of MRSA in healthcare settings remains critical, with a recent study reporting a 45.4% MRSA prevalence among *S. aureus* cases in a maternity and children’s hospital^6^. Meanwhile another tertiary hospital reported a stable MRSA prevalence of approximately 41.2% during 2020, despite disruptions caused by the COVID-19 pandemic^7^. Such statistics emphasize the importance of continuous monitoring of MRSA within healthcare facilities to enhance patient safety and reduce infection rates. Moreover, the growing prevalence of community-associated MRSA ST5 and ST6 in Riyadh’s healthcare facilities raises significant concerns about patient-to-patient transmission and infection control^8^. Epidemiological data have revealed a high incidence of healthcare-associated infections (HAIs) involving MRSA, especially in intensive care units, prompting the need for targeted infection control measures^9^. Additionally, studies have highlighted the alarming prevalence of multidrug-resistant strains, particularly those harboring the Panton-Valentine leukocidin (PVL) gene, which is linked to severe clinical outcomes^10,11^.

Notably, the decline of the pandemic clone CC8/ST239-III, alongside the emergence of new clones, highlights a shifting resistance landscape that continues to pose a significant threat to public health^12,13^. Despite these developments, the genomic features, resistance mechanisms, and clonal relationships of the increasingly dominant ST5 and ST6 clones in Saudi Arabia remain poorly understood^4,5,8^. A clearer understanding of their evolutionary dynamics is essential for developing tailored infection control practices and antimicrobial stewardship efforts.

Previous investigations of MRSA in Saudi Arabia have reported a shifting epidemiology, where older clones such as ST239 and ST80 were historically dominant, while more recent studies have noted increasing detection of community associated lineages including ST5, ST6, ST22, and ST88 across different regions. However, national genomic surveillance remains limited, and most published datasets include small numbers of isolates, underscoring the need for broader genomic characterization to understand current MRSA population structure.

Considering the ongoing challenges posed by MRSA, this study aimed to investigate the emergence and expansion of MRSA ST5 and ST6 within hospitals in Riyadh. By elucidating the genetic characteristics and transmission dynamics of these prevalent strains, we aimed to contribute valuable insights that can effectively inform public health strategies and enhance patient outcomes in the ongoing fight against antibiotic-resistant pathogens.

## Results

### Epidemiological characteristics of MRSA isolates

All 81 MRSA isolates included in this study underwent phenotypic confirmation. Among these isolates, 55 were confirmed by VITEK2, with 54 testing positive on the cefoxitin screen and having an oxacillin MIC ≥ 4 mg/L, while one isolate was cefoxitin-negative with an oxacillin MIC of 0.5 mg/L. Additionally, 26 MRSA isolates were confirmed using BD Phoenix, all of which had cefoxitin MIC ≥ 8 mg/L and oxacillin MIC > 2 mg/L. The distribution of MRSA clones, stratified by age group and infection type, showed significant variations in sequence type prevalence (Table 1). The 31–50 age group had the highest number of MRSA infections, with ST5 and ST6 predominating in respiratory and wound infections. In the younger age group (0–12 years), ST5 and ST6 were notably prevalent in wound, systemic, and respiratory tract infections. The (0–1 years) and (31–50 years) age groups exhibited a diverse range of clones across various infection types, with notable representations of ST88 in the younger age group and ST22 in the older age group, along with prominent ST5, ST6 and other clone representations in different infection types. Statistical analysis indicated significant differences in the prevalence of MRSA clones across age groups (*p* < 0.05), highlighting the importance of demographic factors in understanding MRSA transmission dynamics.

**Table 1:** Distribution of MRSA clones by age group and infection type.

Age group	Infection type	Sequence types (STs)	Count	Percentage (%)
0–1 Year	Wound	6, 88, 1153, 5, 672, 97, 5, 2538	10	12.3
	Systemic	6, 5, 30	5	6.1
	Respiratory	88, 5, 6, 30	4	4.9
2–12 Years	Wound	5, 8, 672, 88	6	7.4
	Respiratory	6, 88, 1535	3	3.7
	Systemic	5	1	1.2
13–17 Years	Respiratory	5	1	1.2
18–30 Years	Respiratory	5, 6, 672, 88	6	7.4
	Wound	22	2	2.4
	Urine	88	1	1.2
	Systemic	5	1	1.2
31–50 Years	Wound	88, 152, 6, 80, 30, 1, 8110, 772, 97, 22, 8	12	14.8
	Respiratory	5, 152, 22, 6, 1930	7	8.6
	Systemic	8111	1	1.2
	Urine	1	1	1.2
51–70 Years	Wound	672, 97, 5, 149, 22, 6	9	11.1
	Systemic	22, 6, 5	5	6.1
	Respiratory	5, 88	2	2.4
	Urine	6	1	1.2
71+ Years	Systemic	97	2	2.4
	Respiratory	88	1	1.2

### Distribution of MRSA clones (MLST)

**Fig. 1:**
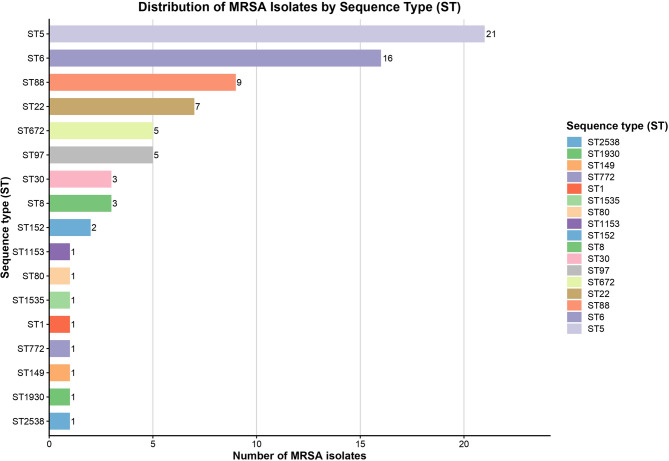
Distribution of MRSA isolates by sequence type based on MLST analysis.

Multi locus sequence typing (MLST) analysis identified 19 distinct STs among the 81 MRSA isolates. The analysis showed that ST5 and ST6 were highly prevalent among MRSA isolates obtained during the study. ST5 accounted for 21/81 (25.9%), while ST6 contributed 16/81 (19.7%). The distribution of ST5 and ST6 among MRSA sequence types (*p* < 0.001) was significantly different from that of other MRSA clones. ST88 and ST22 accounted for 9/81 (11.1%) and 7/81 (8.6%), respectively. Other sequence types had a prevalence of 5/81 (6.1%), including ST672 and ST97, while ST30 and ST8 each represented 3/81 (3.7%). ST152 was identified in 2/81 isolates (2.4%), and the remaining sequence types appeared as singletons (Fig. 1).

### SCC*mec* and spa typing

The analysis of MRSA isolates revealed a diverse distribution of Staphylococcal cassette chromosome *mec* (SCC*mec*) types and Staphylococcal protein A (*spa*) types (Table 2). The most common SCC*mec* type was IVa, representing 51.2% (41/80). Within SCC*mec* IVa, the predominant STs were ST6, ST88, and ST22, which were mainly associated with spa types t304 and t690, indicating limited spa diversity within this SCC*mec* background. SCC*mec* type V was the second most frequent, accounting for 21.2% (17/80). This group showed greater *spa* variability, with ST672 commonly linked to t3841 and ST5 associated with t311. SCC*mec* type VI, which comprised 7.5% (6/80), included ST5 and ST30 and was characterized by *spa* types t688 and t018.

**Table 2:** Distribution of MRSA isolates by SCC*mec* type, sequence type, and spa type.

SCCmec type	ST	Count	spa types (count)
IVa	6	16	t304 (14), t8657 (1), t1627 (1)
	88	9	t690 (5), t2177 (1), t12438 (1), t4494 (1), t2649 (1)
	22	7	t309 (3), t005 (4)
	30	3	t021(2), t018 (1)
	8	3	t008 (3)
	149	1	t045 (1)
	8111	1	t9736 (1)
	1930	1	t4570 (1)
Total*		41	
V	672	5	t3841 (5)
	5	4	t311 (4)
	97	3	t267 (2), t2297 (1)
	1	1	t127 (1)
	1535	1	t084 (1)
	772	1	t345 (1)
	1153	1	t903 (1)
	152	1	t4019 (1)
Total		17	
Vc	5	10	t311 (8), t319 (1), Not typeable (1)**
	2538	1	t3778 (1)
Total		11	
VI	5	5	t688 (5)
	30	1	t018 (1)
Total		6	
IVc	5	2	t105 (2)
	97	1	t521 (1)
	80	1	Not typable (1)
Total		4	
Unclassified***	152	1	t355 (1)
Total		1	

*Total counts represent the cumulative number of isolates identified for each category.

**The spa type for this isolate could not be determined.

***The “Unclassified” category includes isolates with unidentified or unknown SCCmec types.

Less common SCC*mec* types included Vc (11/80, 13.7%) and IVc (4/80, 5%). Most SCC*mec* Vc isolates were ST5 and carried *spa* type t311, although one isolate lacked a resolvable *spa* type. One ST152 isolate remained unclassified for SCC*mec*. Overall, *spa* typing revealed moderate diversity across the MRSA population, with t304, t690, t311, and t3841 being the most common *spa* types. These *spa* types were distributed across wound, respiratory, and systemic specimens without a clear specimen-specific pattern, reflecting their circulation across multiple clinical sources.

### Molecular relationships among MRSA clones (MST analysis)

**Fig. 2:**
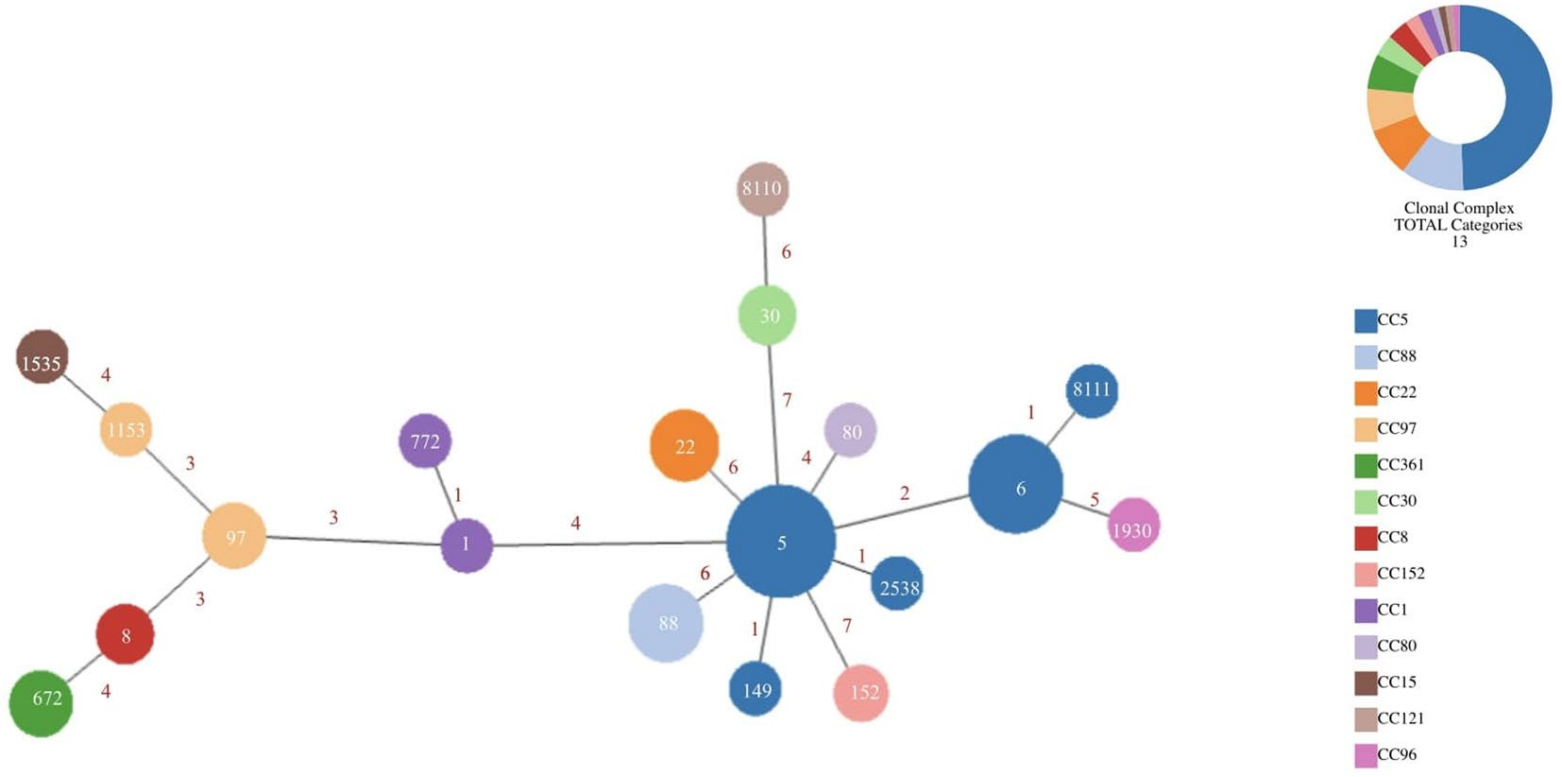
Minimum spanning tree (MST) showing clonal relationships among *S. aureus* sequence types (STs) based on MLST. Node sizes reflect isolate counts, colors indicate CCs, and numbers on lines represent allelic differences. The inset pie chart shows the distribution of isolates by CC.

The minimum spanning tree (MST) analysis of *S. aureus* STs based on MLST data, revealed the genetic relationships among 19 distinct *S. aureus* STs identified in 81 MRSA isolates (Fig. 2). The MST analysis identified ST5 and ST6 (CC5) as the most central and highly connected sequence types in the ST network, comprising 45.6% (37/81) of all MRSA isolates. ST149 differs from ST5 by a single allele (*tpi*), whereas ST6 differs from ST5 by two alleles (*arc*C and *yqi*L); therefore, ST149 is more closely related to ST5 than ST6 within the MRSA/CC5 STs. Meanwhile, ST8111, a newly emerging ST, differs from ST6 by a single allele (*yqi*L), indicating its close evolutionary relationship with ST6.

The genomic relationships among MRSA isolates revealed distinct clonal lineages. ST88 (CC88) and ST22 (CC22) are the most common after ST5, differing by six alleles, indicating genetic divergence. Other notable lineages include ST97 (CC97), a livestock-associated clone, which clusters with ST8 (CC8) and shows moderate clustering with ST672 (CC361), differing by four alleles. Additionally, ST30 (CC30) was identified as a separate lineage, while ST8110, a newly emerged ST, and ST1930 are positioned outside major clonal complexes. These findings highlight the genetic diversity within the MRSA population.

### Genotypic antimicrobial resistance and virulence profiles

Both ST5 and ST6 demonstrated high resistance to methicillin, with ST5 exhibiting a broader antimicrobial resistance profile (Table 3). The presence of resistance genes such as *mecA* and *bla*Z was more frequent in ST5, contributing to its increased resistance compared to that of ST6. Statistical analysis revealed significant differences between these STs in resistance to non-beta-lactam antibiotics (*p* < 0.01).

**Table 3:** Distribution of sequence types (STs) and antimicrobial gene profiles among clonal complexes (CCs) of MRSA isolates.

Clonal complex (CC)	Sequence type (STs)	Antimicrobial gene profile	Frequency (numbers)	Percentage (%)
CC5	5	*mecA*, *bla*Z, *dfr*G, *fos*B-Saur, *fus*C, *tet*(38)	11	13.5
		*mec*A, *bla*Z, *dfr*G, fexA, *fos*B-Saur, *fus*C, *tet*(38), *tet*(M)	5	6.1
		*mec*A, *bla*Z, *dfr*G, *erm*C, *fos*B-Saur, *fus*C, *tet*(38)	2	2.4
		*mec*A, *bla*Z, *dfr*G, *fos*B-Saur, *tet*(38)	2	2.4
		*mec*A, *bla*Z, *dfr*G, fexA, *fos*B-Saur, *fus*C, *tet*(38), *tet*(L)	1	1.2
	6	*mec*A, *fos*B-Saur, *tet*(38)	10	12.3
		*mec*A, *erm*C, *fos*B-Saur, *tet*(38)	3	3.7
		*mec*A, *bla*Z, *fos*B-Saur, *tet*(38)	1	1.2
		*mec*A, ant(6)-Ia, aph(3’)-IIIa, *fos*B-Saur, *msr*A, *sat*4, *tet*(38)	1	1.2
		*mec*A, ant(4’)-Ia, *bla*Z, *erm*C, *fos*B-Saur, *inu*A, *tet*(38), *tet*(K)	1	1.2
	149	*mec*A, *fos*B-Saur, *tet*(38)	1	1.2
	2538	*mec*A, *bla*Z, *dfr*G, *erm*C, *fos*B-Saur, *fus*C, *tet*(38)	1	1.2
	Total	–	39	48.1
CC88	88	*mec*A, *bla*Z, *erm*C, *fus*C, *tet*(38)	4	4.9
		*mec*A, *bla*Z, *fus*C, *tet*(38), *tet*(K)	1	1.2
		*mec*A, *bla*Z, *erm*C, *fus*C, *tet*(38), *tet*(K)	1	1.2
		*mec*A, *bla*Z, *fos*B-Saur, *tet*(38), *tet*(K)	1	1.2
		*mec*A, *bla*Z, *tet*(38), *tet*(K)	1	1.2
		*mec*A, *fos*B-Saur, *fus*C, *tet*(38)	1	1.2
	Total	–	9	11.1
CC22	22	*mec*A, aph(2’’)-Ih, *bla*Z, *dfr*C, *tet*(38)	4	4.9
		*mec*A, aph(2’’)-Ih, *bla*Z, *dfr*C, *erm*C, *tet*(38)	3	3.7
	Total	–	7	8.6
CC361	672	*mec*A, *fos*B-Saur, *fus*C, *tet*(38)	4	4.9
		*mec*A, *erm*C, *fos*B-Saur, *fus*C, *tet*(38)	1	1.2
	Total	–	5	6.1
CC97	97	*mec*A, *fus*C, *tet*(38)	2	2.4
		*mec*A, aph(2’’)-Ih, *fus*C, *tet*(38)	2	2.4
		*mec*A, *tet*(38)	1	1.2
	1153	*mec*A, aph(2’’)-Ih, *fus*C, *tet*(38)	1	1.2
	Total	–	6	7.4
CC30	30	*mec*A, *fos*B-Saur, *fus*C, *tet*(38)	1	1.2
		*mec*A, *fos*B-Saur, *inu*A, *tet*(38)	1	1.2
		*mec*A, *bla*Z, *fos*B-Saur, *inu*A, *tet*(38)	1	1.2
	Total	–	3	3.7
CC8	8	*mec*A, aph(3’)-IIIa, *bla*Z, *fos*B-Saur, mphC, *msr*A, *sat*4, *tet*(38)	3	3.7
CC152	152	*mec*A, aph(2’’)-Ih, *fus*C, *tet*(38)	2	2.4
CC1	1	*mec*A, aph(2’’)-Ih, aph(3’)-IIIa, *bla*Z, *fus*C, *tet*(38), *tet*(L)	1	1.2
	772	*mec*A, aph(3’)-IIIa, *bla*Z, *dfr*G, *fos*B-Saur, mphC, *msr*A, *sat*4, *tet*(38), *tet* (K)	1	1.2
	Total	–	2	2.4
CC80	80	*mec*A, *bla*Z, *fus*B, *tet*(38)	1	1.2
CC15	1535	*mec*A, ant(4’)-Ia, aph(2’’)-Ih, *bla*Z, *fos*B-Saur, *fus*C, *inu*A, *tet*(38)	1	1.2
CC96	1930	*mec*A, *bla*Z, *erm*C, *tet*(38)	1	1.2
Not assigned	8110	*mec*A, *bla*Z, *fos*B-Saur, *tet*(38)	1	1.2
Not assigned	8111	*mec*A, *erm*C, *fos*B-Saur, *tet*(38)	1	1.2
Total			81	100

**Fig. 3:**
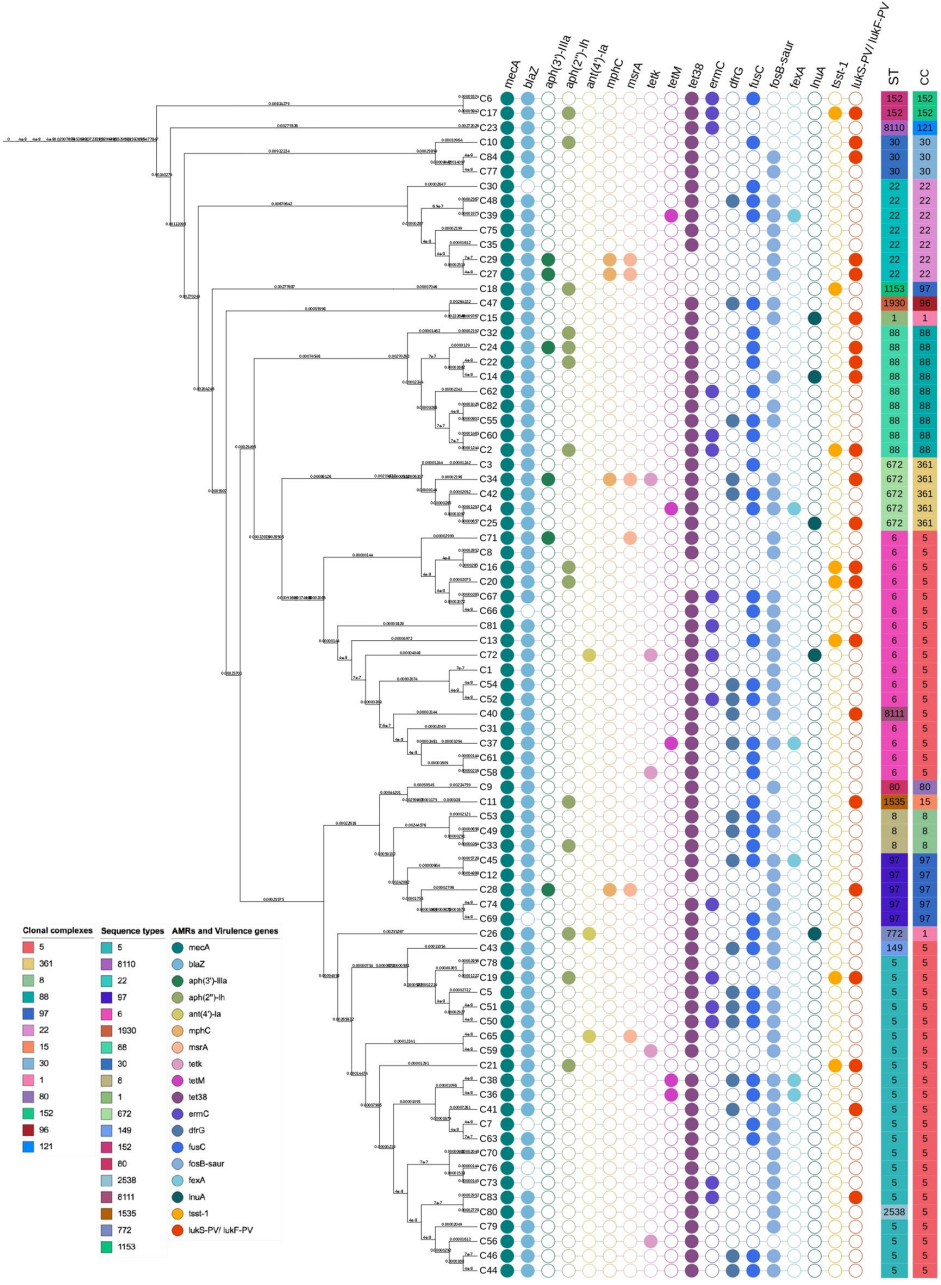
Phylogenetic tree of 81 MRSA isolates showing the relationship between sequence types (STs), clonal complexes (CCs), and the distribution of antibiotic resistance and virulence genes. Isolates are labeled (C1–C84). The presence of resistance genes and virulence factors is indicated alongside each isolate.

The phylogenetic analysis of 81 MRSA isolates reveals a diverse distribution of antibiotic resistance genes (ARGs) and virulence factors (Fig. 3). Key resistance determinants include *mecA* (conferring methicillin resistance), *bla*Z (β-lactam resistance), *erm*C (macrolide resistance), and *tet*K (tetracycline resistance), among others like *aph*(3’)-IIIa, *aph*(2’’)-Ih, *ant*(4’)-Ia, *mph*C, *msr*A, *tet*M, *tet*38, *dfr*G, *fus*C, *fos*B-saur, *fex*A, and *lnu*A. Virulence genes such as *tsst*-1(toxic shock syndrome gene) and *luk*S-PV/*luk*F-PV show variable presence among the STs. The PVL genes (*luk*S-PV/*luk*F-PV) were detected in only three isolates belonging to non-CC5 lineages such as ST88 and ST30. The *tsst*-1 gene was identified in five isolates, predominantly within ST5. In contrast, adhesion-related genes such as *fnb*A were present in nearly all isolates, while enterotoxin genes (*sea* and *seb*) and hemolysin genes (*hla* and *hlb*) showed moderate to low frequencies across different STs. Overall, virulence gene carriage was limited compared to antibiotic resistance determinants.

The analysis also revealed that certain ARGs were associated with specific STs. ST5 isolates consistently carried *mecA* and *blaZ*, along with frequent occurrences of *ermC*, *tetM*, and aminoglycoside resistance genes (*aph(3’)-IIIa*, *aph(2’’)-Ih*, *ant(4’)-Ia*). This suggests that ST5 represents a multidrug-resistant lineage with strong resistance to *β*-lactams, macrolides, and aminoglycosides. In contrast, ST6 isolates also carried *mecA* and *blaZ* but exhibited a lower prevalence of *tetM*. Despite this, they maintained resistance genes against *β*-lactams and aminoglycosides. Unlike ST5, ST6 displayed greater heterogeneity in the presence of ARGs; certain isolates, such as those belonging to ST22 and ST30, lacked some resistance genes, and the frequency of *fusC* and *fosB-saur* was reduced in these clones. Notably, the ST672 clone appeared to have a particularly high prevalence of resistance genes, harboring multiple ARGs, including *mecA*, *blaZ*, *ermC*, *tetK*, *fusC*, and *fosB-saur*. This suggests that ST672 may represent an epidemiologically relevant clone with extensive antibiotic resistance.

### Correlation between ARGs and drug resistance

**Fig. 4:**
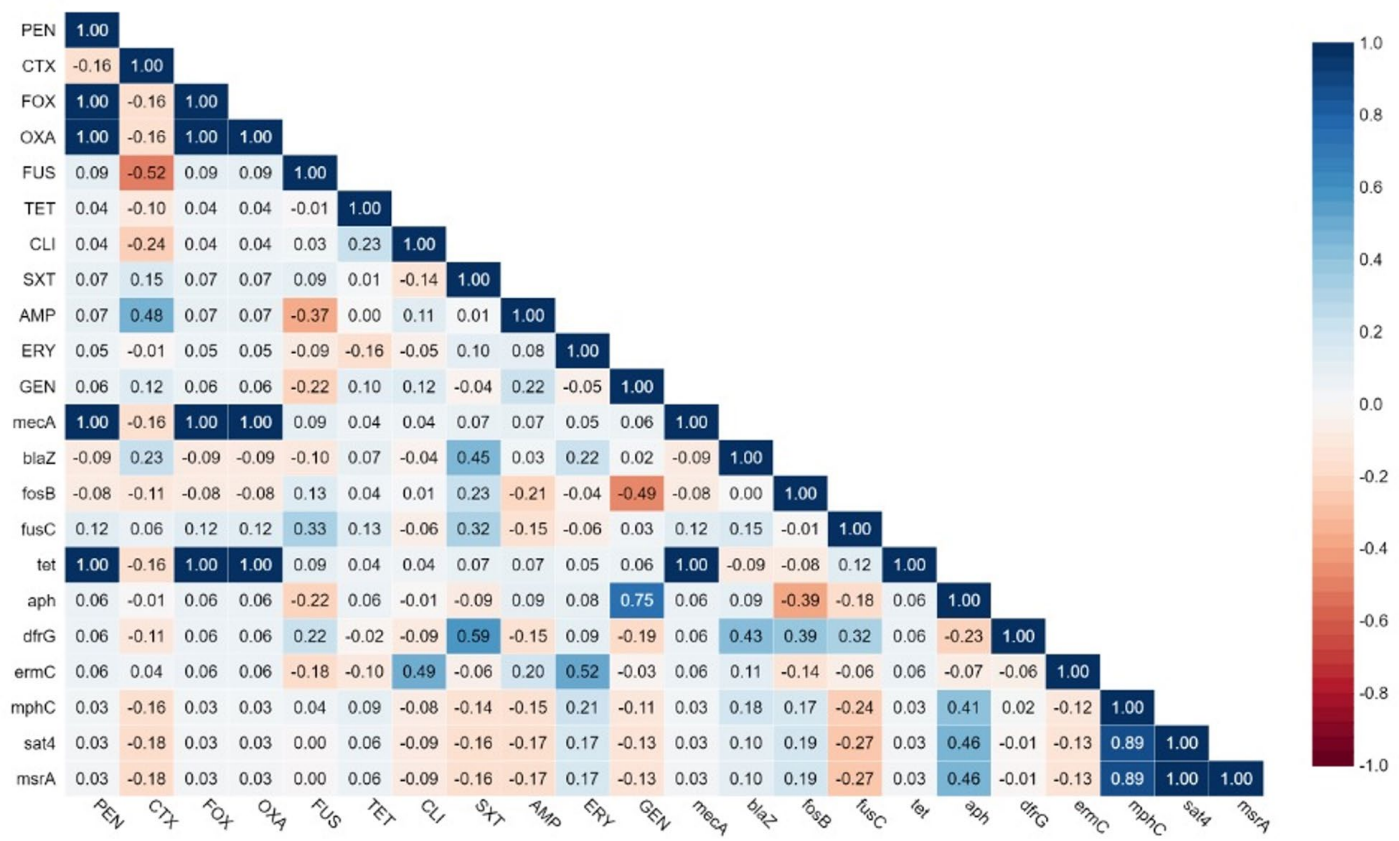
Correlation matrix illustrating the relationships between antimicrobial resistance genes (ARGs) and corresponding antibiotics in MRSA isolates. The color gradient and numerical values represent the strength and direction of correlations, providing insights into co-resistance patterns and the genetic basis of antimicrobial resistance.

The correlation analysis between antimicrobial resistance genes and their corresponding antibiotics in MRSA isolates exhibited varying degrees of association (Fig. 4). For example, strong positive correlations were observed between *mecA* and penicillin (PEN), cefoxitin (FOX), and oxacillin (OXA) (*R* = 1.0), consistent with its well-established role in *β*-lactam resistance. Additionally, *erm*C demonstrated a moderate correlation with erythromycin (ERY) (*R* = 0.52) and clindamycin (CLI) (*R* = 0.49), reinforcing its involvement in macrolide-lincosamide resistance. A high correlation was also observed between *dfr*G and trimethoprim-sulfamethoxazole (SXT) (*R* = 0.59), consistent with its role in trimethoprim resistance. Moreover, *aph* exhibited a strong correlation with gentamicin (GEN) (*R* = 0.75), indicating aminoglycoside resistance. Another notable correlation was observed between *bla*Z and SXT (*R* = 0.45), suggesting potential co-resistance *mec*hanisms. The correlation matrix further revealed possible co-resistance patterns, such as the association between *mecA* and multiple *β*-lactam antibiotics, indicating that the presence of *mecA* may contribute towards a broader resistance profile. Overall, this study indicates that the genetic determinants of antibiotic resistance in multidrug-resistant MRSA isolates are suggested by statistically significant associations (*p* < 0.05) between the identified genetic markers and resistance phenotypes.

## Discussion

This study provides a comprehensive genomic and phenotypic characterization of MRSA isolates circulating in Riyadh healthcare facilities, revealing important insights into the evolving epidemiology of antimicrobial resistance in Saudi Arabia. The rise of ST5 and ST6 highlights their strong adaptation to hospital environments under antibiotic pressure and improved adaptation mechanisms. This represents a notable transition from previous reports of ST239-III dominance in Saudi hospitals^14^ and mirrors global trends where traditional pandemic lineages are being replaced by genetically distinct clones^15^.

MRSA clones ST5 and ST6 are globally significant due to their multidrug resistance and virulence. Originating from the acquisition of *SCCmec* elements (types I–III) carrying the *mecA* gene (conferring *β*-lactam resistance), these clones exhibited broad antibiotic resistance via genes such as *ermB* (macrolide-lincosamide resistance), *msrA* (macrolide efflux), *blaZ* (penicillin resistance), and *tetM* (tetracycline resistance), often acquired through horizontal gene transfer^16–18^. Their dissemination is achieved through key virulence factors, including PVL (a cytolytic toxin that causes necrotizing infections), TSST-1 (induction of systemic shock), fibronectin-binding protein (FnBP; mediates host cell adhesion), and enterotoxins (SEA/SEB; triggers food poisoning and immune hyperactivation)^19^. ST5 is predominantly healthcare-associated, spreading via hospital outbreaks due to its strong biofilm formation and resistance to disinfectants, while ST6 is linked to community transmission in crowded settings^20^. Molecular typing methods (MLST, PFGE, *spa* typing, WGS) revealed their clonal evolution and zoonotic potential, emphasizing the need for integrated One Health strategies^21^.

The genomic architecture of these predominant clones in this study reveals distinct evolutionary pathways that likely reflect different adaptation strategies to hospital environments. ST5’s multidrug resistance suggests high selective pressure, reinforcing its role as a dominant healthcare clone. This multidrug-resistant phenotype aligns with ST-5’s well-established characterization as a pandemic HA-MRSA clone^22^. The significant link between ST5 and hospital-acquired infections (OR: 3.2 for isolates with ≥ 4 resistance genes) reflects its evolutionary adaptation to hospital settings, where antibiotic pressure and vulnerable patients favor multidrug-resistant strains^23^. This finding is consistent with reports from other Middle Eastern countries documenting the increasing prevalence of ST5 in critical care settings^24^.

In contrast to ST5 clone, ST6 demonstrated a more variable resistance pattern that suggests a different evolutionary history. While like ST5 clone, universal *mecA* presence (100%) is maintained, it showed significantly lower prevalence of *bla*Z (18.7% compared to 90.2% in ST5) but a higher prevalence of aminoglycoside resistance genes. This resistance profile, combined with its frequent detection in pediatric and general-ward patients, suggests that ST6 may have originated in community settings before adapting to hospital environments; a phenomenon increasingly recognized worldwide^25^. Higher *fos*B-Saur in ST6 may reflect local fosfomycin use and clone-specific adaptation^26^.

The microevolution of these clones within CC5 was highlighted by the MST analysis. ST149 and ST8111 indicate ongoing microevolution, signaling potential future emerging variants of interest. These findings are supported by similar reports from other Gulf Cooperation Council (GCC) countries documenting the emergence of region-specific variants through point mutations and genetic recombination^5,27,28^. The detection of these variants is particularly important as they may represent early stages in the development of new epidemic clones with potentially enhanced virulence or resistance properties.

Many genotype-phenotype associations with clinical and epidemiological consequences were identified via the correlation matrix analysis. The correlations between *mecA* and resistance *β*-lactams (*R* = 1.0 for all) confirms this genetic determinant’s pivotal involvement in MRSA development and supports its ongoing application as a major diagnostic marker^29^. However, some discordance was observed between resistance patterns that warrants further investigation. Most notably, while *tet* 38 was present in all the MRSA isolates, its correlation with tetracycline resistance was weaker than expected, suggesting the involvement of additional regulatory mechanisms or alternative resistance pathways that may modulate phenotypic expression.

The resistance gene profiles showed several clone-specific patterns with important clinical implications. ST5 isolates demonstrated a high prevalence of *erm*C (65.8%), which showed moderate correlation with resistance to erythromycin (*R* = 0.52) and clindamycin (*R* = 0.49). This was particularly relevant for antimicrobial stewardship programs, as these antibiotics are commonly used for MRSA treatment^30^. The co-occurrence of *erm*C and *msr*A in certain ST6 isolates may have contributed to the considerably lower correlation values compared to those reported from European populations (typically *R* > 0.7), which can make resistance predictions more difficult^31,32^. Another finding was the strong association between *aph* genes and gentamicin resistance (*R* = 0.75), which exceeded correlation values typically observed in Western MRSA populations (*R* ~ 0.6) and likely reflects region-specific aminoglycoside usage patterns^33,34^.

One of the most important results of this study was the moderate correlation (*R* = 0.45) between *bla*Z and trimethoprim-sulfamethoxazole resistance. While these resistance mechanisms are not biochemically linked, this association may indicate co-localization of resistance determinants on mobile genetic elements, as previously observed in Middle Eastern EMRSA-15 isolates^5,35^. Such arrangements can facilitate the co-selection of resistance traits under antibiotic pressure, potentially accelerating the development of multidrug resistance^36^. This phenomenon needs attention in antimicrobial stewardship programs, as it suggests that use of one antibiotic class could simultaneously select for resistance to unrelated agents.

The virulence gene distribution patterns revealed important differences between the predominant clones. While ST5 exclusively carried *tsst*-1, both ST5 and ST6 lacked the PVL genes that are characteristic of many community-associated MRSA strains. This contrasts sharply with the PVL positivity rates observed in ST88 (20%) and ST30 (33%) isolates in our study and aligns with previous reports from Saudi pediatric populations^6^. The presence of *tsst*-1 gene in ST5 is particularly concerning given this clone’s association with critical infections, as the toxin can cause life-threatening toxic shock syndrome in vulnerable patients. These findings suggest that different MRSA clones may occupy different ecological niches within healthcare facilities, with varying pathogenic potentials that could influence patient health outcomes.

On the other hand, the emergence of recent clones, such as ST672, as clones of potential concerns warrant special attention. For example, MRSA ST672 isolates carried an exceptionally high number of resistance genes (seven or more per isolate), including *bla*Z, *erm*C, and *tet*(K), demonstrating a resemblance to multidrug-resistant strains recently reported from India^37^. While currently representing a small proportion of isolates (6.1%), the genetic flexibility demonstrated by this clone suggests it may have significant potential for future expansion, particularly if selective antibiotic pressures continue.

Overall, our findings highlight key implications for MRSA control in Saudi healthcare settings. The dominance of multidrug-resistant ST5 in hospitals calls for enhanced surveillance and targeted precautions, while ST6’s presence in both hospital and community settings suggests that integrated transmission tracking is required. High antibiotic resistance genes such as, *bla*Z, *tet*, and *fos*B-Saur prevalence are examples of region-specific resistance patterns that suggest the necessity of local antibiotic management. Emerging clones (ST672) and novel variations (ST8111) highlight the importance of continuous genomic monitoring. One limitation was the study’s focus on a single region (Riyadh city hospitals, and regional laboratories) and potential undetected resistance mechanisms. Additionally, the study design was cross-sectional, and longitudinal genomic surveillance would be needed to confirm clonal dynamics over time. Further research is needed to clarify transmission routes and guide targeted interventions against clinically significant clones such as ST5 and ST6.

## Conclusion

This study provides comprehensive genomic and epidemiological insights into the emergence and dominance of MRSA ST5 and ST6 in Riyadh’s healthcare facilities. Key findings include the high prevalence of ST5 in ICUs and its multidrug-resistant phenotype, the broader dissemination of ST6 in general hospital wards, and the active clonal diversification within CC5. Strong gene-to-phenotype correlations highlight the complexity of co-resistance patterns, complicating treatment and containment efforts. The evolutionary replacement of older clones like ST239 with more adaptive ones such as ST5 and ST6 highlights the evolving epidemiological landscape of MRSA. Future directions should focus on long term genomic surveillance, clone-specific infection control interventions, and the identification of environmental reservoirs to reduce transmission. Integrated strategies leveraging molecular epidemiology, clinical data, and public health measures are essential for mitigating the burden of MRSA in Saudi Arabia and beyond.

## Methods

### Sample collection and isolation

Between February and June 2022, 81 non-duplicate MRSA isolates were collected from human specimens at the Riyadh Regional Laboratory and Blood Bank.

Each isolate originated from a unique clinical specimen obtained from a different patient; no repeat samples were included. The specimens included wound swabs, blood cultures, respiratory tract samples, and sterile body fluids. Patients included both males and females across pediatric and adult age groups who presented for routine diagnostic testing. The identification of MRSA was performed using the VITEK2 system (bioMérieux, Marcy-L’Etoile, France) and BD Phoenix (BD Diagnostics, Franklin Lakes, NJ, USA). All samples were subjected to microbiological analyses within 24 h of collection.

### Identification and confirmation of *S. aureus* and MRSA

The *S. aureus* isolates were cultured on self-prepared sheep blood agar plates using Columbia blood agar powder (OXOID, Basingstoke, UK), and the clinically isolated samples showing β-hemolysis were compared with the ATCC 25,923 reference strain (ATCC, Manassas, VA, USA). Confirmatory tests were conducted on isolated colonies using mannitol salt agar (MSA; Neogen, Lansing, MI, USA), Gram staining, catalase, and coagulase tests (PROLEX™, Pro-Lab Diagnostics, Richmond hill, ON, Canada). MRSA detection was performed using Harlequin MRSA Chromogenic Agar supplemented with cefoxitin (Neogen). Positive identification was indicated by the presence of blue colonies, which are characteristic of MRSA, following 24 h of incubation. Following initial confirmation of *S. aureus* and MRSA identity through culture-based methods, isolates underwent molecular testing to detect the *mecA* gene, thereby ensuring both phenotypic and genotypic validation of resistance.

### PCR-based *mec*A detection

DNA was extracted from pure bacterial colonies utilizing the Qiagen DNeasy Blood and Tissue Kit (Qiagen, Hilden, Germany), according to the manufacturer’s instructions. DNA purity was assessed using a QIAxpert Spectrophotometer (Qiagen), and DNA concentration was quantified with a Qubit™ Flex Fluorometer (Thermofisher Scientific, Waltham, MA, USA). The *mecA* gene, which encodes penicillin-binding protein 2a (PBP2a), was targeted to confirm methicillin resistance. A 310-bp fragment was amplified using specific primers: forward primer 5′-GTAGAAATGACTGAACGTCCGATAA-3′ and reverse primer 5′-CCAATTCCACATTGTTTCCGTAA-3′ both synthesized by Macrogen (Seoul, South Korea)^38,39^. The PCR reaction included DreamTaq™ 2X Green PCR Master Mix (Thermofisher Scientific), primers at a concentration of 10 pmol, and 2 µL of DNA template, achieving a total volume of 25 µL. The positive MRSA control used was *S. aureus* ATCC 43,300 (ATCC). The amplification process involved an initial denaturation step at 95 °C for 4 min, followed by 30 cycles of denaturation at 95 °C for 45 s, annealing at 56 °C for 1 min, and extension at 72 °C for 1 min, concluding with a final extension at 72 °C for 4 min. The annealing temperature of 56 °C was optimized to ensure specific binding of the primers to the *mecA* gene, thereby minimizing non-specific amplification. The resulting PCR product was analyzed through electrophoresis on 1.6% agarose gel stained with ethidium bromide, and visualized using Syngene image analysis software (Syngene, Bangalore, India).

### WGS and bioinformatics analysis

WGS libraries were prepared using a QIAseqFX DNA library preparation kit (Qiagen), with 100 ng of DNA as input, in accordance with the manufacturer’s protocols. Libraries were selected for a 300–350 bp insert size. Sequencing was conducted on a NovaSeq 6000 platform (Illumina, San Diego, CA, USA) across two SP lanes. Data quality control was executed, applying a Phred score cutoff of Q30 to ensure high-quality sequencing data with a base call accuracy of 99.9%. Bioinformatics analyses were performed using the Bactopia V2 pipeline, tailored specifically to *S. aureus* workflows (https://github.com/bactopia/bactopia)^39^. The CARD and ResFinder databases were employed for the detection of resistance markers, while screening for PVL was conducted using the local BLAST database (accessed in December 2023).

### Statistical analysis

Data analysis was conducted using GraphPad Prism version 10.0.0 software (GraphPad Software, Boston, MA, USA). The significance of differences was evaluated using the chi-square (χ²) test in a two-tailed format, with *p* < 0.05 considered statistically significant. A correlation matrix comprising Pearson’s correlation coefficients was calculated using the “corrplot” package in R version 4.2.1^40^.

## Data Availability

The datasets generated and analyzed during the current study are available in the European Nucleotide Archive (ENA) under the accession number PRJEB64197 and can be accessed at https://www.ebi.ac.uk/ena/browser/view/PRJEB64197.
